# Canine Distemper Virus in Asiatic Lions of Gujarat State, India

**DOI:** 10.3201/eid2511.190120

**Published:** 2019-11

**Authors:** Devendra T. Mourya, Pragya D. Yadav, Sreelekshmy Mohandas, R.F. Kadiwar, M.K. Vala, Akshay K. Saxena, Anita Shete-Aich, Nivedita Gupta, P. Purushothama, Rima R. Sahay, Raman R. Gangakhedkar, Shri C.K. Mishra, Balram Bhargava

**Affiliations:** Indian Council of Medical Research, National Institute of Virology, Pune, India (D.T. Mourya, P.D. Yadav, S. Mohandas, A. Shete-Aich, R.R. Sahay);; Sakkarbaug Zoo, Junagadh, India (R.F. Kadiwar, M.K. Vala);; Department of Principal Chief Conservator of Forest, Gandhinagar (A.K. Saxena, P. Purushothama);; Indian Council of Medical Research, New Delhi (N. Gupta, R.R. Gangakhedkar, B. Bhargava);; Ministry of Environment, Forest and Climate Change, New Delhi, India (S.C.K. Mishra)

**Keywords:** Canine distemper virus, Asiatic lion, next-generation sequencing, RT-PCR, heminested PCR, India, Gir National Park, viruses

## Abstract

In September 2018, an epizootic infection caused by canine distemper virus emerged in an Asiatic lion population in India. We detected the virus in samples from 68 lions and 6 leopards by reverse transcription PCR. Whole-genome sequencing analysis demonstrated the virus strain is similar to the proposed India-1/Asia-5 strain.

Canine distemper virus (CDV; genus *Morbillivirus*) causes highly contagious disease in a wide range of carnivores. Epizootic disease in lions in a wildlife sanctuary in California, USA, in 1992 and Serengeti National Park, Tanzania, in 1994 underlined the potential of CDV to cause fatality in wild felids (*1*,*2*). The disease often manifests as respiratory and gastrointestinal signs that progress to neurologic disease (*2*).

A single isolated population of Asiatic lions (*Panthera leo persica*) resides in the Gir forests of Gujarat State, India, the last natural habitat for this species. Conservation efforts brought this lion population back from the brink of extinction and increased their numbers (*3*). 

During 2 weeks in September 2018, the unusual death of 28 lions of all age groups was reported from Gir Wildlife Sanctuary. A detailed investigation revealed 18 additional lions exhibited dullness, dehydration, lacrimation, cough, diarrhea, and seizures. Necropsy of 2 carcasses showed edema and purulent exudates in the lungs. Histopathology of lungs from both lions showed mononuclear cell infiltration with mild thickening of interalveolar septa. 

The Indian Council of Medical Research, National Institute of Virology (Pune, India), received ocular, nasal, and rectal swab specimens packed in a viral transport medium and blood samples from 229 wild and 87 captive lions and visceral organs, including lung, liver, heart, and kidney, from 3 dead lions for virologic investigation. Of the 229 wild lions, 20 showed clinical signs, including dullness, lacrimation, cough, diarrhea, and seizures; 2 of the 87 captive lions showed lacrimation and respiratory distress. 

We extracted RNA by using Magmax Total RNA Isolation Kit (ThermoFisher Scientific, https://www.thermofisher.com) and processed samples for heminested PCR for CDV and nested PCR for *Paramyxovirus*, as described previously (*4*). We obtained 287 bp by heminested reverse transcription PCR (RT-PCR) and 500 bp by *Paramyxovirus* nested RT-PCR. We detected CDV from >1 sample from 68 (21.3%) lions, including 56 (24.5%) wild and 12 (13.8%) captive lions. All 22 of the lions with clinical signs were PCR-positive for CDV. Among the samples tested, 18/90 (20%) blood, 26/131 (19.8%) rectal, 28/131 (21.4%) nasal, and 10/132 (7.5%) ocular specimens, as well as the visceral organs from the 3 dead lions, were CDV positive. 

We performed RNA library preparation and quantification for next-generation sequencing by using methods described previously (*5*) and analyzed reads by using CLC Genomics Workbench version 11.0.1 (QIAGEN, https://www.qiagen.com). We retrieved the near-complete genome (15 kb) of CDV from 11 lion samples by using a combination of de novo assembly and reference mapping. We calculated percent nucleotide differences by using the p-distance method in MEGA version 7.0 (*6*). We performed similarity and divergence calculations for the H gene and generated a neighbor-joining tree for the complete genome and hemagglutinin (H) gene region. We performed bootstrap replication of 1,000 cycles to assess the robustness of the tree.

Phylogenetic analysis showed that sequences from this outbreak clustered with East-African CDV strains (Appendix Figure 1). The complete genome of CDV among the positive samples displayed 100% similarity, except for the spleen and lung of 1 lion, which were 99.9% similar. We noted a 3.4% nucleotide difference between the complete genome of Gir CDV and the East-African strain (Table). 

**Table:** Nucleotide similarity of the hemagglutinin gene and complete genome of canine distemper virus sequences to those from samples collected from an Asiatic lion, India, 2018*

GenBank accession no.	Source	Geographic location	Year	Lineage	H gene	Complete genome
MK037459	Asiatic lion	Gir National Park, Gujarat State, India	2018		100.0	100.0
MK037460					100.0	100.0
MK037461					100.0	100.0
MK037462					100.0	99.9
MK037463					100.0	99.9
MK037464					100.0	99.9
MK037465					100.0	100.0
MK037466					100.0	100.0
MK037467					100.0	100.0
MK037468					100.0	100.0
KU578257.1	Dog	Africa	1994	East African	96.1	96.6
MH496778.1	Dog	Thailand	2014	Asia 4	94.5	94.1
KJ466106.1	Raccoon dog	China	2012	Asia 1	94.0	94.1
AY649446.1	Racoon	USA	2001	America 2	94.9	94.9
AY466011.2	Racoon	USA	1998	America 1	93.0	94.0
AY386316.1	Dog	Germany	NA	Europe	95.9	94.8
AB475099.1	Dog	Japan	NA	Asia 2	93.4	94.1
KC479141.1	Dog	India	2012		97.1	NA
KC479140.1	Tiger	India	2012		96.9	NA
KC479139.1	Tiger	India	2012		97.1	NA
KC479138.1	Red panda	India	2013		97.4	NA
LC011103.1	Dog	India	2012		97.4	NA
Vaccine strains						
AF378705.1 CDV strain Onderstepoort					92.4	92.1
EU726268.1 CDV strain CDV3					92.6	92.0
Z35493.1CDV Convac					92.0	NA

*Reference sample is GenBank accession no. MK037469. NA, not available. GenBank accession nos. MK037459–68 represent sequence data from this study.

Phylogenetic analysis of the CDV sequences of Gir lions against available partial CDV sequences of H gene previously collected from tigers and a dog from India demonstrated a distinct cluster for sequences from India (Appendix Figure 2). Bhatt et al. (*7*) hypothesized a novel CDV strain, India-1/Asia-5, among domestic dogs in India. Our analysis of sequences from the CDV outbreak in Gir lions strengthens that hypothesis. We observed 95.8%–96.8% nucleotide similarity for the H gene region (Table) of CDV sequences from the Gir outbreak with the proposed India-1/Asia-5 strain. We noted minimal nucleotide similarity between Gir CDV outbreak strains and Asia 3 strains and maximum similarity with Rockborne-like strains. Whole-genome sequence analysis showed that Gir CDV strains had ≈8% nucleotide difference from the 2 known vaccine-derived strains of America 1 genotypes (Table).

After we confirmed CDV in Asiatic lions, samples were collected from other wild animals in Gir Wildlife Sanctuary. We detected CDV in 6/52 (12%) samples from *Panthera pardus* leopards but not in samples from *Viverricula indica* civets, *Panthera tigris* tigers, *Canis lupus pallipes* wolves, or *Caracal caracal* cats.

Before 2019, few instances of CDV were reported in lions, tigers, red pandas, and leopards from zoos and forests in India. However, canine distemper is prevalent among dogs in India (*8*), and the free-ranging dog population often poses a threat of CDV transmission to wildlife (*9*). Other wildlife species also could play a role in maintenance and transmission of CDV (*9*). Vaccination is an option; attenuated Onderstepoort strain was used successfully in captive African lions in Maasai Mara National Reserve in Kenya, and a live attenuated canine vaccine was used in a vaccine trial in tigers (*10*). Reintegration of the existing lion population from the Gir region to different sanctuaries can ensure the protection and conservation of the species.

Appendix Additional information on detection of canine distemper virus in Asiatic lions of Gujarat, India.
