# Supplementary material for: Canine Distemper Virus in Asiatic Lions of Gujarat State, India
Source: Emerg Infect Dis. 2019 Nov;25(11):2128–30. doi: 10.3201/eid2511.190120 (PMC6810198; doi:10.3201/eid2511.190120)
Supplement: Appendix — Additional information on detection of canine distemper virus in Asiatic lions of Gujarat, India. [file 19-0120-Techapp-s1.pdf]

Article DOI: https://doi.org/10.3201/eid2511.190120

Canine Distemper Virus in Asiatic Lions of Gujarat State, India

Appendix

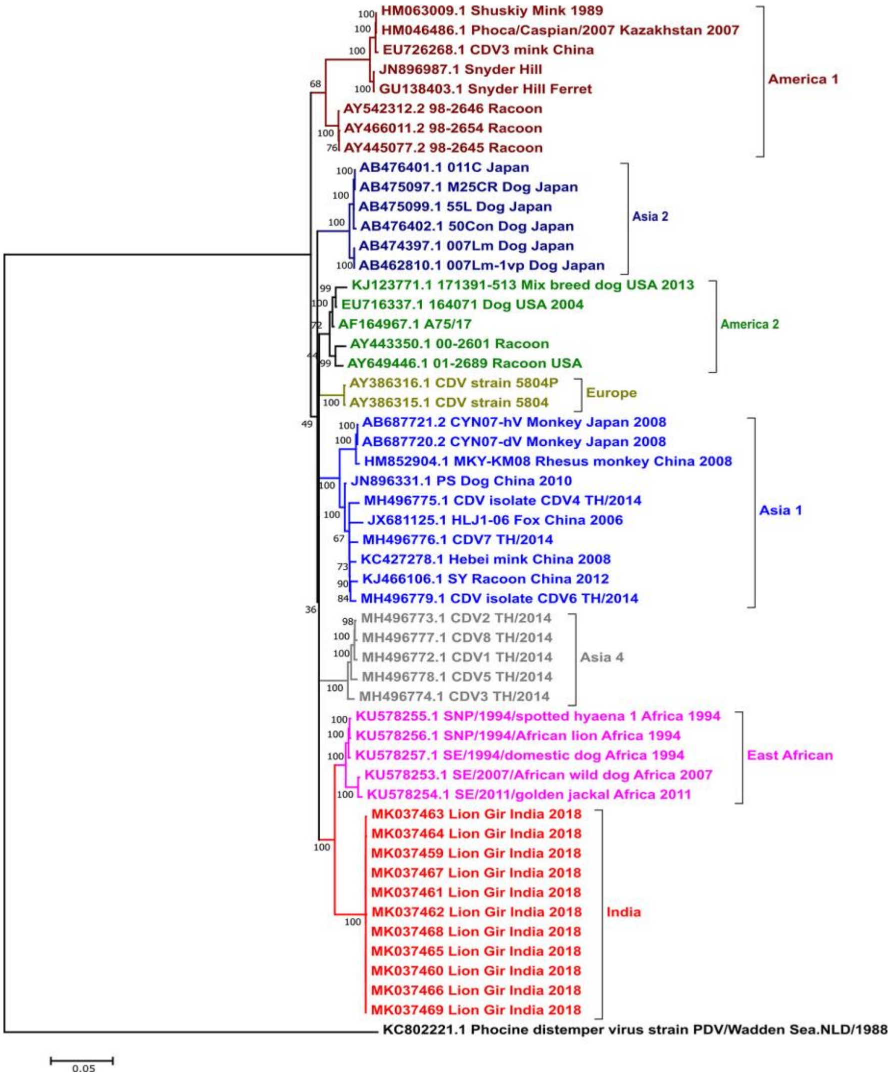

Appendix Figure 1. Phylogenetic analysis of the complete genome of canine distemper virus from samples collected from Asiatic lions, Gir National Forest, India, 2018. A maximum-likelihood method along with GTR + gamma + I model was used to generate the tree. Different colors represent different lineages of canine distemper virus. Red text denotes sequences from the reported outbreak. Pink text represents sequences reported from other outbreaks in India. Scale bar represents nucleotide substitutions per site.

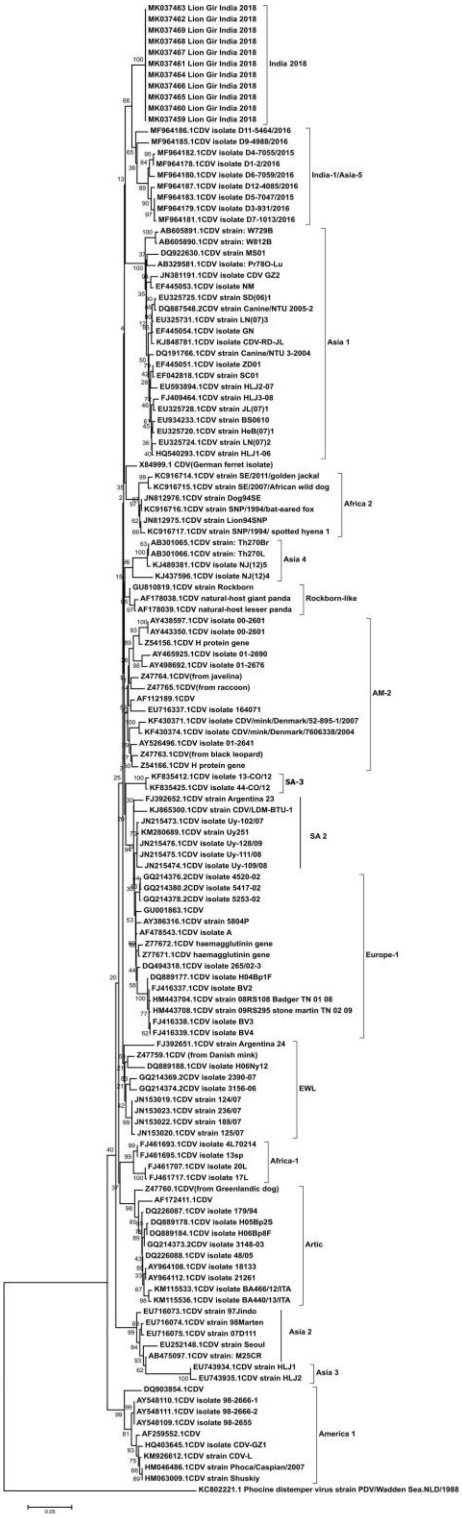

Appendix Figure 2. Phylogenetic analysis of the H gene from canine distemper virus from samples collected from Asiatic lions, Gir National Forest, India, 2018. A maximum-likelihood method along with Tamura 3 parameter + gamma model was used to generate the 688 bp tree. Scale bar represents nucleotide substitutions per site.
